# Molecular mechanisms of thalidomide effectiveness on COVID-19 patients explained: ACE2 is a new ΔNp63α target gene

**DOI:** 10.1007/s00109-024-02485-x

**Published:** 2024-09-18

**Authors:** Laura Monteonofrio, Ilaria Virdia, Sara Pozzi, Roberto Quadri, Alessandra Amendolare, Flaviana Marzano, Micaela Braile, Virginia Sulfaro, Moira Paroni, Apollonia Tullo, Silvia Soddu, Luisa Guerrini

**Affiliations:** 1 Dipartimento Di Ricerca E Tecnologie Avanzate, Istituto Nazionale Tumori Regina Elena IRCCS, 00144 Rome, Italy; 2 Dipartimento Di Bioscienze, Università Degli Studi Di Milano, Via Celoria 26, 20133 Milan, Italy; 3 Istituto Di Biomembrane, Bioenergetica E Biotecnologie Molecolari (IBIOM), Consiglio Nazionale Delle Ricerche, 70025 Bari, Italy

**Keywords:** Thalidomide, SARS-CoV-2, ACE2, ΔNp63α, COVID 19

## Abstract

**Abstract:**

COVID-19 pandemic is caused by the SARS-CoV-2 virus, whose internalization and infection are mediated by the angiotensin-converting enzyme 2 (ACE2). The identification of novel approaches to tackle this step is instrumental for the development of therapies for the management of COVID-19 and other diseases with a similar mechanism of infection. Thalidomide, a drug sadly known for its teratogenic effects, has potent immunomodulatory and anti-inflammatory properties. Treatment with this drug has been shown to improve the immune functions of COVID-19 patients and proposed for the management of COVID-19 in clinical practice through drug repositioning. Here, we investigated the molecular details linking thalidomide to ACE2 and COVID-19, showing that in conditions mimicking SARS-CoV-2-associated cytokine storm, the transcription factor ΔNp63α and ACE2 are stabilized, and IL-8 production is increased. In such conditions, we found p63 to bind to and regulate the expression of the *ACE2* gene. We previously showed that ΔNp63α is degraded upon thalidomide treatment and now found that treatment with this drug—or with its analogue lenalidomide—downregulates ACE2 in a p63-dependent manner. Finally, we found that thalidomide treatment reduces in vitro infection by pseudo-SARS-CoV-2, a baculovirus pseudotyped with the SARS-CoV-2 spike protein. Overall, we propose the dual effect of thalidomide in reducing SARS-CoV-2 viral re-entry and inflammation through p63 degradation to weaken SARS-CoV-2 entry into host cells and mitigate lung inflammation, making it a valuable option in clinical management of COVID-19.

**Key messages:**

- Thalidomide treatment results in p63-dependent ACE2 downregulation.
- ACE2 is a p63 transcriptional target.
- Thalidomide reduces the “cytokine storm” associated to COVID-19.
- Thalidomide prevents viral re-entry of SARS-CoV-2 by p63-dependent ACE2 downregulation.
- Thalidomide is a modulator of SARS-CoV-2 or other ACE2-dependent infections.
- ACE2 is modulated by a pharmacological substance.

## Introduction

SARS-CoV-2 infection, mediated by the binding of the viral spike (S) protein to the ACE2 receptor in the host cells [1–3], causes the COVID-19 disease. One of the associated complications is a cytokine storm which involves an uncontrolled release of cytokines and chemokines, leading to a systemic inflammatory response linked to lung failure and acute respiratory distress syndrome. Given the time and cost involved in developing new therapies, repurposing existing drugs with known profiles has become a cost-effective strategy [4]. Therefore, reassessing the efficacy of licensed and experimental drugs has been a go-on choice by the World Health Organization (WHO) and other health agencies to treat emerging health problems. With the initial lack of vaccines and effective agents against SARS-CoV-2, as well as public health emergencies, WHO had identified promising reposition therapies, such as the combination of two anti-HIV drugs (i.e., lopinavir and ritonavir) or the experimental antiviral compound, remdesivir [5]. Furthermore, thalidomide (Thal), a small molecule drug with many years of history known to cause misery, became a game changer for its multifaceted pharmacological effects such as immunomodulation, anti-inflammation, anti-angiogenesis, and antiviral effects [6, 7]. Several Thal derivatives have been designed, known as immunomodulatory drugs (IMids), among which the most used are lenalidomide (Len) and pomalidomide, that are approved by FDA for the treatment of several severe diseases like multiple myeloma, myelodysplastic syndrome (5q-), mantle cell lymphoma, and follicular lymphoma [8]. IMids retain the anti-angiogenic and anti-inflammatory properties of Thal but are devoid of Thal teratogenic activity [6, 8].

Extensive information available on Thal’s mechanisms of action and its efficacy and safety in hemophagocytic syndrome–induced cytokine storm, idiopathic pulmonary fibrosis (IPF), or severe H1N1 and paraquat poisoning lung injury argues for the possible action of Thal on COVID-19-induced lung effects and cytokine storm [9–12]. Recent reviews on the COVID-19 treatment endorse the possibility of usage of Thal and its analogues for treating COVID-19 patients. Thal, sadly known for its teratogenic action [13], has been shown to have potent immunomodulatory/anti-inflammatory activities [14], to prevent the development of bleomycin-induced pulmonary fibrosis in mice by blocking the TGF-β1 pathway [9], and to improve respiratory symptoms and life quality in patients with IPF [10, 11] or H1N1 infection [12]. Based on these observations, a repositioning study with Thal, in COVID-19 patients, has been performed in China with positive results [15]. Moreover, Len at very high doses has been shown to induce ACE2 downmodulation through a post-translational mechanism [16].

We have long studied the molecular basis of Thal’s teratogenic effects, specifically its role in degrading ΔNp63α and TAp63α proteins through the ubiquitin ligase CRL4 cereblon (CRBN) [17]. The p63 proteins are members of the p53 family of transcription factors and consist of ten isoforms with distinct and important biological roles during development [18, 19]. In epithelial cells, including lung epithelial cells, the ΔNp63α isoform is the most prominent one in regulating proliferation, apoptosis, and differentiation [20]. In addition, ΔNp63α regulates the transcription of several cytokines, such as IL-1α [21], IL-31, IL-33 [22], and IFN-γ [23], and is expressed in lung epithelium [24]. Based on these observations, we reasoned that the beneficial effects upon Thal treatment observed in COVID-19 patients might be related to ΔNp63α degradation with subsequent modulation of its transcriptional targets. These targets would include already-known factors, such as inflammatory cytokines and novel targets such as the SARS-CoV-2 entry receptor ACE2.

## Materials and methods

### Cell culture, treatments, and transfection

A431 (human epidermoid cell line; ATCC# CCL-1555) and A549 (human lung carcinoma cell line; ATCC# CCL-185) were kindly provided by Dr. Ada Sacchi (Istituto Nazionale Tumori Regina Elena, Rome, Italy), H1299 (human lung carcinoma cell line; ATCC# CRL-5803) was kindly provided by Dr. Giovanni Blandino (Istituto Nazionale Tumori Regina Elena, Rome, Italy), U-2 OS (human osteosarcoma cell line; ATCC# HTB-96) was kindly provided by Prof. Francesco Blasi (Università degli Studi di Milano, Milan, Italy), and HaCaT (human keratinocyte cell line) was kindly provided by Prof. Antonio Costanzo (Humanitas Research Hospital, Milan, Italy). All cells were maintained in DMEM enriched with 10% fetal bovine serum (Euroclone #ECS0165L), 1 mM l-glutamine (Euroclone #ECB3000D), 100 units/mL penicillin, and 100 μg/mL streptomycin (Euroclone #ECB3001D) at 37 °C in a humidified atmosphere of 5% (v/v) CO_2_. Cells were maintained in culture for no more than ten passages and underwent routine testing to ensure that they are mycoplasma-free. For treatments with thalidomide (Tocris #50–35-1) and lenalidomide (LGC products #191,732–72-6), 5 × 10^4^ cells were seeded onto 24-well multi-plates and 20 h later incubated with different drug concentrations and times indicated in the figure legends. For transient transfection, 5 × 10^4^ A431 cells were plated in 24-well multi-plates and on the next day transfected with Lipofectamine 2000 (Invitrogen #11,668,019) with increasing amount of CEREBLON (CRBN) encoding plasmid or with four different p63-small hairpin RNA (sh-p63) vectors with sequence homology to four different regions of p63 mRNA (OriGene Technologies # TF308688); shRNA-SCRAMBLED (sh-SCRB) was used as a control. For stable transfection of A431 cells, shp63#3/shp63#4 and shSCRB plasmids were transfected with Lipofectamine 2000 (Invitrogen #11668019), and after 2 days, 1 μg/mL puromycin was added to select stable transfectants. The cells were maintained as polyclonal populations under puromycin at 0.25 μg/mL. For U-2 OS, 5 × 10^4^ cells were plated in 24-well multi-plates and the next day transfected with Lipofectamine 2000 with increasing amount of ΔNp63α encoding plasmid.

### Western blot (WB) analysis

At the indicated times, cells were lysed in 100 μL of Loading Buffer 2X (2% sodium dodecyl sulfate, 30% glycerol, 144 mM β-mercaptoethanol, 100 mM Tris–HCl pH 6.8, and 0.1% bromophenol blue). Samples were incubated at 98 °C for 10 min and resolved by SDS-PAGE. Proteins were transferred to a nitrocellulose membrane (Amersham #GEH10600001). The blots were incubated with the following antibodies (Abs): anti-p63 4A4 (Santa Cruz Biotechnology sc-8431), anti-ACE2 (Abcam ab15348), anti-CRBN (Cell Signaling Technology D8H3S), and anti-actin (Santa Cruz Biotechnology sc-8432). The following secondary Abs were used: goat anti-mouse IgG-HRP (Santa Cruz Biotechnology sc-2005) and goat anti-rabbit IgG-HRP (Santa Cruz Biotechnology sc-2030). Proteins were visualized by an enhanced chemiluminescence method (GeneSpin #STSE500) according to the manufacturer’s instructions using a ChemiDoc Touch (BioRad).

### Plasmids

The plasmids carrying ΔNp63α, CRBN, or shCRBN were previously described [17].

### RNA purification, reverse transcriptase RT-PCR, and quantitative real-time qPCR analyses

Total mRNA from cells was isolated using the RNeasy miniKit (Qiagen #74,104). cDNA was synthesized by M-MLV RTase and amplified with GoTaq DNA polymerase (Promega #M3001). For quantitative PCR analysis, mRNA expression level was evaluated using the Power SYBR Green PCR Master Mix with ABI Prism 7500HT Fast Real-Time PCR System Detector (Applied Biosystems). Relative mRNA expression levels were determined by using the 2-∆∆CT method, employing GAPDH gene expression for data normalization. All reactions were performed in triplicate. Primer sequences are as follows:

ACE2 forward 5′-CAT TGG AGC AAG TGT TGG ATC TT-3′;

ACE2 reverse 5′-GAG CTA ATG CAT GCC ATT CTC A-3′;

GAPDH forward 5′-TCC CTG AGC TGA ACG GGA AG-3′;

GAPDH reverse 5′-GGA GGA GTG GGT GTC GCT GT-3′.

### ELISA assay

HaCaT cells, 1.8 × 10^5^, were plated in 35-mm culture dishes and treated with recombinant human TNF-α (rhTNF-α; BioLegend # BMS301) 5 ng/mL for 7 or 17 h or with 0.5 µg/mL LPS from *E. coli* (SIGMA #O111:B4) for 4, 6, or 17 h. Quantification of IL-8 in the supernatants of treated HaCaT cells was performed by ELISA (hIL-8; ImmunoTools #31,670,089) according to the manufacturer’s instructions. The ELISA plates were read by a microplate reader (SAFAS MP96).

### Chromatin immunoprecipitation (ChIP)

A431 cells, 4 × 10^6^, were plated in 15-cm culture dishes and treated with LPS 0.5 µg/mL for 2 h. Then, proteins were cross-linked to DNA in living nuclei and the ChIP assay was performed using the MAGnify™ Chromatin Immunoprecipitation System (Thermo Fisher #492,024), as described by the manufacturer. The following quantities of Abs were used to immunoprecipitate the DNA–protein complexes: 2 μg of anti-p63α (D2K8X) XP® Rabbit mAb (Cell Signaling Technology #131,095), 2 μg of anti-acetylated H4-histone Ab (BioRad #AHP148), and 1 μg of an unrelated, negative control Ab. DNA fragments obtained by the ChIP assay were analyzed by qPCR using specific primers spanning the responsive elements (RE) found in the first intron of the *ACE2* gene. Primers specific for exon 8 of the *ACE2* gene were used as the negative control. As a positive control for PCR and as normalizer, DNA prepared from samples prior to immunoprecipitation (whole cell lysates) was used as total or input DNA. Primer sequences are as follows:

First intron of the *ACE2* gene (+ 1067; + 1087):

Forward: 5′-ACGACGCGTGTGGAGAAGTCCATCAGA-3′;

Reverse: 5′-ACGAGATCTGGTCAACCACACATACCA-3′;

Exon 8 *ACE2* gene (+ 19,634; + 19,730):

Forward: 5′-GGGATGCACAGAGAATATTCAAGG-3′;

Reverse: 5′-AGACTGCTTTCTGAACATTTCCTG-3′.

### In vitro* SARS-CoV-2 spike protein pseudovirus infection*

A baculovirus expressing the green fluorescent protein (GFP) and pseudotyped with SARS-CoV-2 spike protein (Montana Molecular #C1110G) was employed to evaluate its entry into cells through ACE2 receptor. To assess Thal-induced effect, 5 × 10^3^ A431 cells were plated in triplicates in 96-well plates. Thal was added 17 h post cell seeding; after 24 h of treatments, 50 μL of pseudo-SARS-CoV-2 suspension (viral titer: 2 × 10^10^ viral genes (VG) per mL) was added to each well following the manufacturer’s instructions. At 30 h post-infection, cells were washed with PBS, fixed with 4% formaldehyde in PBS for 10 min at room temperature, incubated for 10 min with the reagent containing 4,6-diamidino-2-phenylindole (DAPI), and washed again with PBS. Images were acquired using a Nikon CSU-W1 microscope in widefield mode, using a × 20 objective and analyzed with FIJI [25].

### FACS analysis

For FACS analysis, shp63 and shSCRB stable transfectants were plated and infected with the SARS-CoV-2 spike protein pseudovirus as above, and after 24 h, the FACS analysis was performed by a BD (Franklin Lakes, NJ, USA) FACSCanto II flow cytometer. Data were analyzed with FlowJo software (version 10.4.2; BD, Franklin Lakes, NJ, USA).

## Results

### Thalidomide and lenalidomide at clinically relevant concentrations reduce ACE2 expression

**Fig. 1:**
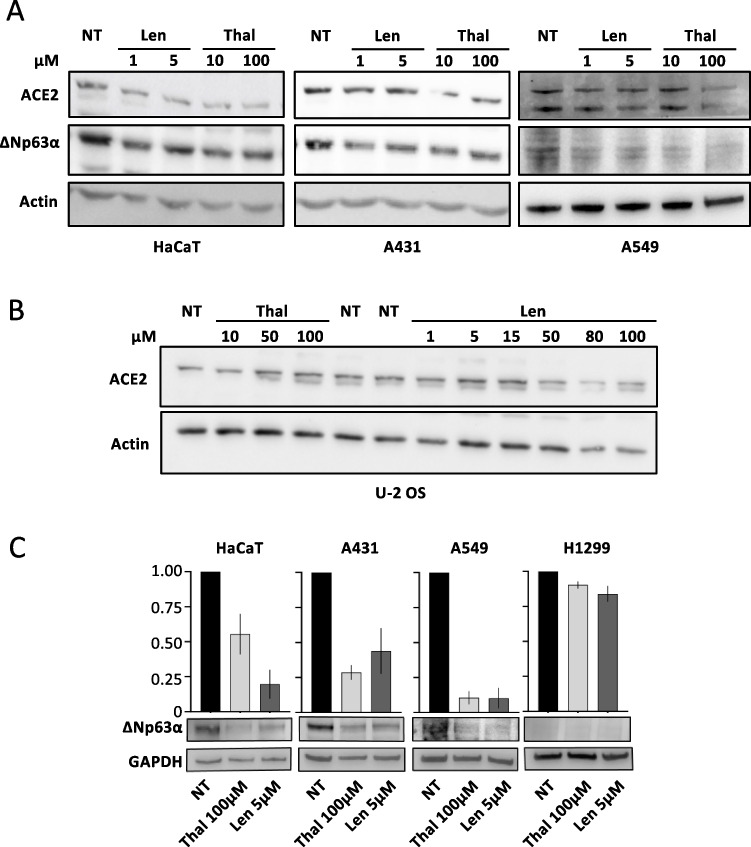
Thal and Len at clinically relevant concentrations reduce ACE2 expression. **A** HaCaT, A431, and A549 cells were treated for 24 h with Len (1 or 5 µM) or Thal (10 or 100 µM). Cell extracts were prepared and analyzed by WB with anti-ACE2 or anti-p63 Abs. Actin was used as the loading control, and one representative experiment is shown. **B** p63-null U-2 OS cells were treated for 24 h with increasing concentrations of Thal (10, 50, 100 µM) or Len (1, 5, 15, 50, 80, 100 µM). Cell extracts were analyzed by WB with anti-ACE2 Ab. Actin was used as the loading control, and one representative experiment is shown. **C** HaCaT, A431, A549, and H1299 cells were treated with Thal 100 µM or Len 5 µM for 8 h; DMSO was used as the control (NT). Lower panel: cell extracts were prepared and analyzed by WB with anti-p63 Ab; GAPDH was used as the loading control, and one representative experiment is shown. Upper panel: compared to the relative Ctrl, ACE2 mRNA levels decreased in p63-proficient HaCaT, and A431 and A459 cells were treated with Thal or Len but not in p63-deficient H1299 cells. ACE2 mRNA was evaluated by qRT-PCR, mRNA levels were normalized using GAPDH, and the means ± standard errors (SE) of three replicates are shown

We first investigated whether Thal or Len could modulate the expression of ACE2 receptor in association with ΔNp63α degradation in human cells, such as HaCaT keratinocytes, A431 epidermoid cells, and A549 lung carcinoma cells, which express both ΔNp63α and ACE2 proteins. Cells were treated for 24 h with 10 or 100 µM Thal or 1 or 5 µM Len. As expected [17], ΔNp63α protein was degraded by the pharmacological treatments, with the ACE2 levels decreasing with the reduction of ΔNp63α levels in the three cell lines (Fig. 1A). We then verified whether Thal or Len could modulate ACE2 expression in p63-null cells in a dose-dependent manner, since it has been reported that Len at high concentrations induces ACE2 downmodulation by a post-translational mechanism [16]. At the highest concentrations of Thal, we did not observe ACE2 downmodulation in the p63-null U-2 OS human osteosarcoma cells. In contrast, the treatment with Len at the high concentrations reported to modulate ACE2 expression [16] (i.e., 80–100 µM) resulted to be toxic, as it can be inferred by the concomitant reduction of the actin levels (Fig. 1B). Next, we assessed whether modulation of ACE2 in response to Thal or Len treatment is regulated also at the transcription level. HaCaT, A431, and A549 cells were treated for 24 h with 100 µM Thal or 5 µM Len and ACE2 mRNA levels measured by qRT-PCR. Human lung carcinoma H1299 cells that do not express any of the p63 isoforms were used as further negative control. In ΔNp63α proficient cells, ΔNp63α protein was degraded by both treatments with a concomitant decrease of ACE2 mRNA levels, whereas we did not observe ACE2 mRNA decrease in the p63-null cells (Fig. 1C).

Taken together, these results suggest that ACE2 could be a ΔNp63α target gene with Thal or Len leading to CRBN-mediated ΔNp63α degradation (CRBN is part of the E3 ubiquitin ligase complex that targets ΔNp63α for degradation upon Thal treatment) [17, 26] that in turn would give rise to reduced ACE2 transcription.

### Thalidomide reduces ACE2 expression through CRBN-mediated ΔNp63α degradation

**Fig. 2:**
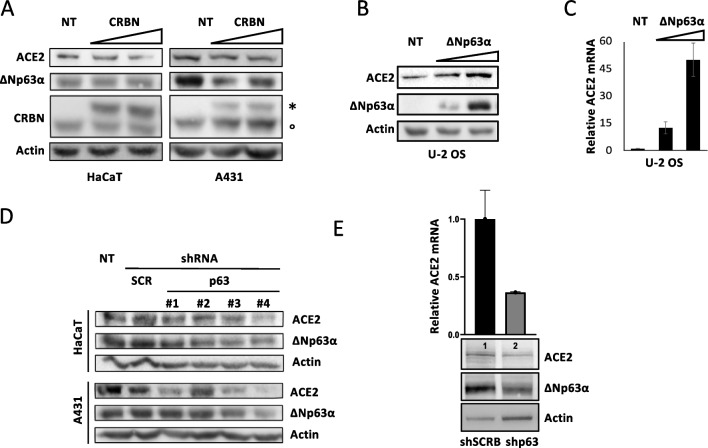
ΔNp63α and ACE2 parallel modulation at both protein and mRNA levels. **A** HaCaT and A431 cells were transiently transfected with 100 and 250 ng of CRBN encoding plasmid. After 24 h from transfection, cell extracts were prepared and analyzed by WB with anti-ACE2, anti-p63, and anti-CRBN Abs. Actin was used as the loading control; one representative experiment is shown. Two bands of the CRBN protein are evident: °stays for endogenous CRBN, *for transfected CRBN that has higher molecular weight due to an HA tag. **B** p63-null U-2 OS cells were transiently transfected with 10 and 25 ng of ΔNp63α expression vector. After 24 h from transfection, cell extracts were prepared and analyzed by WB with anti-ACE2 or anti-p63 Abs. Actin was used as the loading control, and one representative experiment is shown. **C** ACE2 mRNA levels were evaluated by qRT-PCR, and mRNA levels were normalized using GAPDH as housekeeping gene. Means ± SE of three replicates are shown. **D** HaCaT and A431 cells were transiently transfected with 100 ng of small hairpin RNA (shRNA) plasmids targeting p63 mRNA; four different p63 shRNA vectors with sequence homology to four different regions of p63 mRNA were used (#1, #2, #3, #4); the cells were also transfected with a control (100 ng), shRNA-SCR, that encodes for an RNA that is not complementary to any mRNA sequence. After 24 h from transfection, cell extracts were prepared and analyzed by WB with anti-ACE2 and anti-p63 Abs. Actin was used as the loading control; one representative experiment is shown. **E** A431 cells were stably transfected with both p63shRNA#3 and p63shRNA#4, and polyclonal populations were isolated by puromycin selection. As the control, cells were also transfected with a shRNA-SCRB vector. Cell extracts were prepared and analyzed by WB with anti-ACE2 and anti-p63 Abs, and parallel samples were used for ACE2 mRNA level evaluation by qRT-PCR; mRNA levels were normalized using GAPDH as housekeeping gene. Means ± SE of three replicates of three cultures are shown

To verify this hypothesis, we reduced ΔNp63α protein levels by transfecting HaCaT and A431 cells with the CRBN encoding plasmid. As expected [17], CRBN overexpression led to a dose-dependent ΔNp63α degradation in both cell lines (Fig. 2A) that was paralleled, also in this case, by ACE2 downmodulation, supporting the idea that ACE2 levels are correlated with ΔNp63α levels. Next, we transiently transfected the p63-null U-2 OS cells with a ΔNp63α encoding plasmid and observed a positive correlation between expression levels of ACE2 and ΔNp63α (Fig. 2B). Moreover, evaluation of ACE2 mRNA by qRT-PCR in parallel samples showed increased ACE2 mRNA levels in the samples transfected with the ΔNp63α plasmid, thus indicating that ACE2 might be a ΔNp63α transcriptional target (Fig. 2C). To evaluate this point, we performed p63 silencing in HaCaT and A431 cells by transfection with small hairpin RNA (shRNA) plasmids targeting the p63 mRNA. For this type of experiment, we used four different p63 shRNA vectors (OriGene) with sequence homology to four different regions of the p63 mRNA, with the shp63#3 and the shp63#4 vectors resulting in the strongest effect on ΔNp63α silencing and a concomitant decrease of ACE2 protein levels, in both cell lines (Fig. 2D). Since we did not find a wholly consistent matching between ΔNp63α downregulation and reduction of ACE2 protein levels with all shp63 RNAs, we generated stable transfected polyclonal populations with the shp63#3 and shp63#4 and the relative control with the shSCRB vector. Reduced level of ΔNp63α protein was associated with the reduction of ACE2 protein and mRNA levels (Fig. 2E) further supporting the ΔNp63α role in ACE2 regulation.

### ACE2 is a new ΔNp63α target gene

**Fig. 3:**
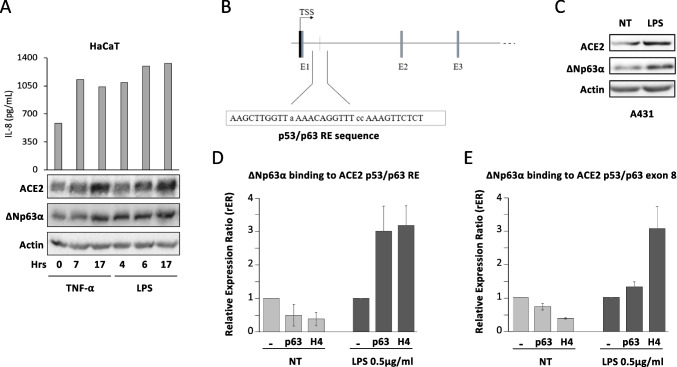
ACE2 is a new ΔNp63α target gene. **A** HaCaT cells were treated with 5 ng/mL of TNF-α or with 0.5 µg/mL of LPS for the indicated times. Cell extracts were prepared and analyzed by WB with an anti-ACE2 and anti-p63 Abs. Actin was used as the loading control, and one representative experiment is shown. The cell supernatants were collected, and IL-8 levels were quantified by the ELISA assay. **B** The schematic representation and sequence of the p53/p63RE were identified in the first intron of *ACE2.* **C** A431 cells were treated or not with LPS 0.5 μg/mL for 2 h. Cell extracts were prepared and analyzed by WB using anti-ACE2 and anti-p63 Abs. Actin was used as the loading control, and one representative experiment is shown. **D**, **E** A431 cells were treated with LPS for 2 h and then collected for chromatin immunoprecipitation (ChIP) analysis

One of the main problems with SARS-CoV-2 infection is the triggering of a “cytokine storm” [27], a hyper-inflammatory state characterized by the production of extremely high levels of proinflammatory cytokines that eventually leads to patient death. In order to mimic the hyper-inflammatory state in vitro, HaCaT cells were treated with LPS or the proinflammatory cytokine TNF-α, both known to stabilize ΔNp63α protein levels and to stimulate cytokine production, possibly by stabilized ΔNp63α acting on the promoter of several cytokine genes [28–30]. Upon TNF-α and LPS treatments, both ΔNp63α and ACE2 expression levels were induced in a dose-dependent and time-dependent manner (Fig. 3A, lower panel). The levels of IL-8, known to be overproduced in COVID-19 patients and expressed in HaCaT cells [31, 32], were also increased by our treatments (Fig. 3A, upper panel), likely by stabilized ΔNp63α.

We then verified whether the p53 family responsive elements (RE) were present in the regulatory regions of the *ACE2* gene. For this purpose, we queried the p53Fam-Tag database [33] and identified one strong putative p53/p63-RE composed by three decamers in the first intron of the *ACE2* gene (Fig. 3B). To evaluate the in vivo recruitment of ΔNp63α on the identified p53/p63-RE, a chromatin immunoprecipitation assay (ChIP) was performed. Cross-linked chromatin from A431 cells treated with LPS 0.5 µg/mL for 2 h was immunoprecipitated with anti-acetylated H4-histone or anti-p63α Abs. In the presence of LPS (i.e., 2 h treatment), but not in the untreated control cells, ΔNp63α was consistently recruited on the p53/p63-RE of the *ACE2* gene (Fig. 3D). The increased p63 occupancy was accompanied by an increase in histone H4 acetylation (Fig. 3D) and, consistently, in ACE2 protein levels (Fig. 3C). As negative control, the exon 8 of the *ACE2* gene, not containing any p53/p63-RE, was not amplified in the same samples (Fig. 3E). Taken together, these results clearly indicate that ACE2 is a new target gene of ΔNp63α.

### Thalidomide weakens in vitro infection by *pseudo*-SARS-CoV-2

**Fig. 4:**
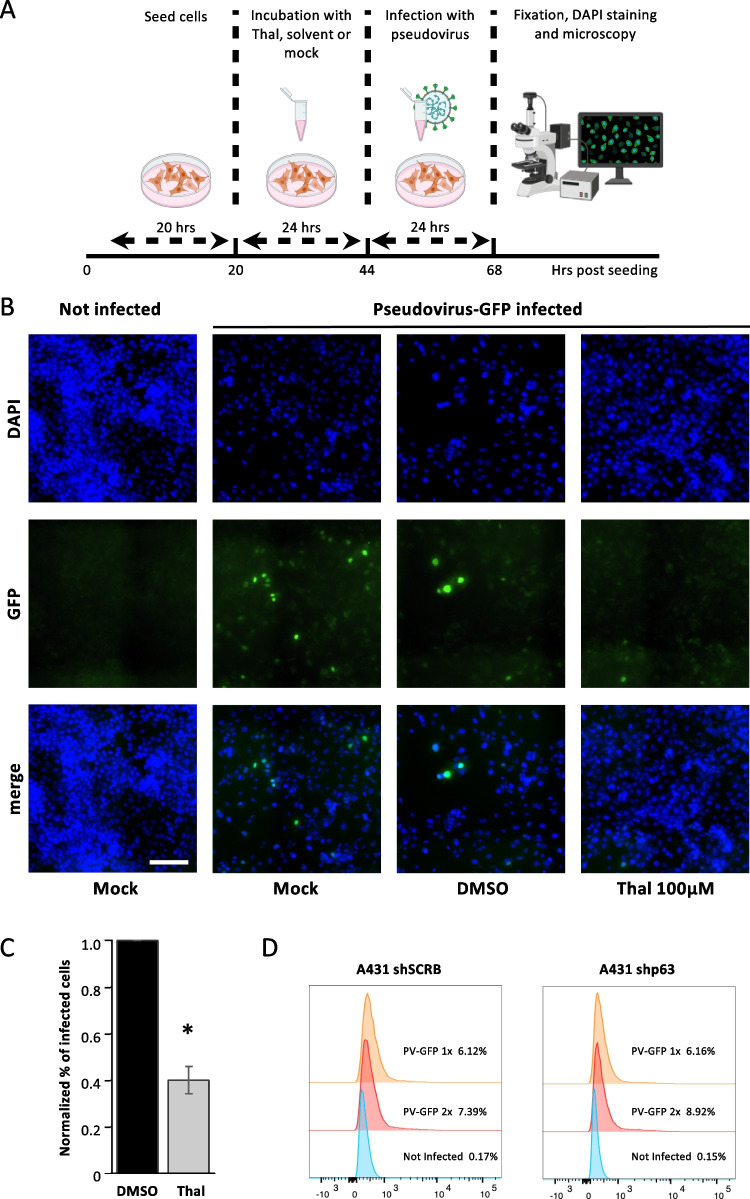
Thal impairs in vitro infection by GFP-expressing pseudo-SARS-CoV-2. **A** The schematic representation of the experiment is shown in **B**. **B** Immunofluorescent staining of A431 cells treated with Thal 100 µM; after 24-h treatment, 50 µL of pseudo-SARS-CoV-2 suspensions was added to the cells following the manufacturer’s instructions; the viral titer was 2 × 10^10^ viral genes (VG) per mL. After 24 h post-infection, cells were washed with PBS and fixed with 4% formaldehyde in 1X PBS for 10 min at room temperature. Nuclei were stained with DAPI. Images were acquired using a Nikon CSU-W1 microscope in widefield mode, using a × 20 objective and analyzed with FIJI [24], and representative images are shown. Scale bar 150 μm. **C** Quantification of GFP reduction in Thal-pretreated samples. **D** FACS analysis was performed 24 h after pseudo-SARS-CoV-2 infection of shp63#3/shp63#4 and shSCRB stable transfectants

It has been reported that COVID-19 patients treated with Thal had a faster recovery in respect to untreated patients [15]. From the data obtained, we hypothesized that the observed protection might be due to the diminished viral re-entry due to ACE2 downmodulation upon Thal treatment as a consequence of ΔNp63α degradation. To verify this hypothesis, we pretreated A431 cells with 100 μM Thal for 24 h before adding, for additional 24 h, the pseudo-SARS-CoV-2, a GFP-expressing baculovirus pseudotyped with the SARS-CoV-2 spike protein (Fig. 4A). We found that Thal pretreatment impairs pseudoviral infection in vitro, as evidenced by reduced GFP signals in the Thal-pretreated samples compared with the controls (Fig. 4B and C). Next, we assessed whether ΔNp63α downregualtion is sufficient to reduce pseudo-SARS-CoV-2 infectivity by employing the stable shp63#3/shp63#4 transfected cells. No difference was observed between control shSCRB and ΔNp63α downregulated cells suggesting that Thal triggers additional events to ΔNp63α degradation to reach its effects (Fig. 4D).

Altogether, these data offer a mechanistic explanation of the protective effect from severe COVID-19 observed in patients treated with Thal [15].

## Discussion

COVID-19 is an infectious disease caused by SARS-CoV-2 that started to spread at the end of 2019. The impellent need for effective therapies at the beginning of the pandemic drove several drug repositioning approaches to tackle the severe adverse effects of the virus infection [5]. Among these, the usage of the immunomodulatory drug Thal was proposed, and its administration proved effective in fastening patient recovery and reducing the levels of inflammatory cytokines in the serum of the patients [15].

Thal was originally used as anti-emetic compound to treat morning sickness of pregnant women and was withdrawn from the market in 1961 due to its teratogenic effects [6, 13]. This adverse effect was recently shown to depend on the Thal-induced degradation of ΔNp63α and TAp63a, two isoforms of the p63 transcription factor known to have essential roles in skin and limb development [17]. Nonetheless, this drug has found usage in clinical practice due to its anti-tumoral, immunomodulatory, and anti-inflammatory effects [7, 34]. In addition, it has been observed that the SARS-CoV-2 virus induced lower mortality in Len-treated multiple myeloma patients compared to Len-naïve counterparts [35], supporting that Thal and its analogues might be useful in the management of COVID-19 patients. However, the underlying molecular mechanism is still unclear. Starting from these evidences, we hypothesized that the beneficial effects observed in COVID-19 patients treated with Thal could stem from ΔNp63α degradation, with p63 eventually playing a role on the regulation of ACE2. Noteworthy, this p63-mediated mechanism of protection by SARS-CoV-2 infection has been observed in studies exploiting other known inducers of p63 degradation, such as metformin [36, 37]. Supporting this hypothesis, we identified in silico a p53/p63-RE in the *ACE2* gene and demonstrated that, in conditions mimicking the well-known COVID-19-associated cytokine storm, p63 does indeed bind to this element. On top of this, *ACE2* mRNA and protein levels both scale with p63 abundance in the cell, as shown by p63 overexpression or silencing further proving that p63 is indeed active at the *ACE2* locus to promote ACE2 expression. We speculated that the effects observed in COVID-19 patients treated with Thal or Len could likely be caused by a p63 degradation–dependent reduction of ACE2 levels, and that this downmodulation could reduce the SARS-CoV-2 viral infection and, in COVID-19 patients, viral re-entry. This hypothesis holds true as we show that Thal induces ACE2 reduction only in cells expressing p63 and reduces infection by a pseudo-SARS-CoV-2 virus. However, additional factors such as TMPRSS2 [1, 38] have been recently shown to be required, together with ACE2, to allow SARS-CoV-2 infection. The observation that Thal does reduce pseudo-SARS-CoV-2 infectivity, while the sole ΔNp63α downregulation does not, suggests that Thal might regulate multiple factors, warranting further investigations.

Overall, our work sheds new light onto current comprehension of Thal and p63 molecular details at various levels. First, our data envisage new roles of p63 on the regulation of cell protein expression and support the general concept that Thal may efficiently modify the expression of genes through p63 regulation, among which is *ACE2*. Second, they support a protective role for Thal against SARS-CoV-2 viral infection and explain the molecular details of how this compound exerts its functions, potentially making it a valuable option in the management of SARS-CoV-2 or other ACE2-dependent infections. Third, this work represents a proof of concept of a pharmacological substance that may play an effect on ACE2. This effect opens to new consideration on possible roles that the modulation of ACE2 cellular levels may offer in the clinical practice. Indeed, modulation of ACE2 levels with Thal may prevent or effectively contrast SARS-CoV-2 infection if properly given in early phases of the virus attack. While these data suggest a potential new preventive approach to SARS-CoV-2, they also pose some important potential problems related to the use of Thal, the major one being the need to consider the negative effect of ACE2 blockage on the renin–angiotensin–aldosterone system and specifically on hypertension and its effects on heart and renal function. It seems that specific anti-angiotensin II therapies such as ARBs should follow and/or be given in concomitance with Thal. Clinical trials should be designed to resolve this aspect.

## Data Availability

The datasets used and/or analyzed during the current study are available from the corresponding author on reasonable request.
